# The Dimensionality of Hydrogen Bond Networks Induces Diverse Physical Properties of Peptide Crystals

**DOI:** 10.1021/acsmaterialslett.4c00665

**Published:** 2024-07-23

**Authors:** Hui Yuan, Pierre-Andre Cazade, Chengqian Yuan, Bin Xue, Vijay Bhooshan Kumar, Rusen Yang, Gal Finkelstein-Zuta, Lihi Gershon, Maoz Lahav, Sigal Rencus-Lazar, Yi Cao, Davide Levy, Damien Thompson, Ehud Gazit

**Affiliations:** † The Shmunis School of Biomedicine and Cancer Research, George S. Wise Faculty of Life Sciences, Tel Aviv University, Tel Aviv 6997801, Israel; ‡ Department of Physics, Bernal Institute, University of Limerick, Limerick V94 T9PX, Ireland; § State Key Laboratory of Biochemical Engineering, Institute of Process Engineering, Chinese Academy of Sciences, Beijing 100190, China; ∥ National Laboratory of State Microstructure, Department of Physics, Nanjing University, Nanjing 210093, Jiangsu, China; ⊥ Academy of Advanced Interdisciplinary Research, School of Advanced Materials and Nanotechnology, Xidian University, Xi’an 710126, China; # Center for Nanoscience and Nanotechnology, Wolfson Applied Materials Research Center, University of Tel Aviv, Tel Aviv 6997801, Israel

## Abstract

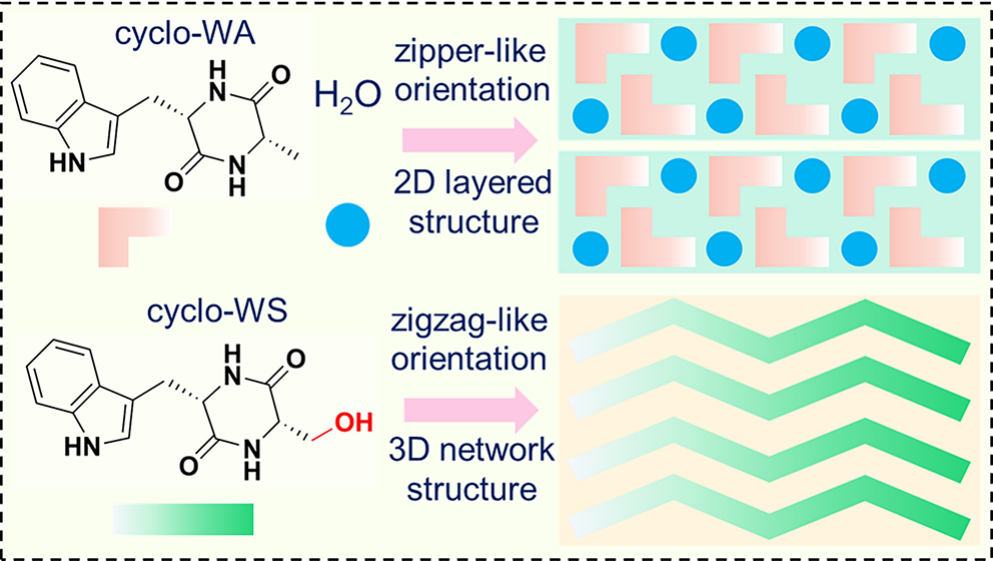

Short peptides are attractive building blocks for the fabrication of self-assembled materials with significant biological, chemical, and physical properties. The microscopic and macroscopic properties of assemblies are usually closely related to the dimensionality of formed hydrogen bond networks. Here, two completely different supramolecular architectures connected by distinct hydrogen bond networks were obtained by simply adding a hydroxyl group to switch from cyclo-tryptophan-alanine (cyclo-WA) to cyclo-tryptophan-serine (cyclo-WS). While hydroxyl-bearing cyclo-WS molecules provided an additional hydrogen bond donor that links to adjacent molecules, forming a rigid three-dimensional network, cyclo-WA arranged into a water-mediated zipper-like structure with a softer two-dimensional layer template. This subtle alteration resulted in a 14-fold enhancement of Young’s modulus values in cyclo-WS compared to cyclo-WA. Both cyclo-dipeptides exhibit biocompatibility, high fluorescence, and piezoelectricity. The demonstrated role of dimensionality of hydrogen bond networks opens new avenues for rational design of materials with precise morphologies and customizable properties for bioelectronic applications.

Short peptides and their bioinspired derivatives serve as promising building blocks for myriad potential applications of supramolecular assemblies at the interface of life sciences and nanotechnology.^1−6^ Peptides can self-assemble into supramolecular structures and architectures with long-range ordered arrangements governed by noncovalent interactions that can be controlled to embed valuable characteristics, including mechanical, piezoelectric, optical, and electronic properties.^7,8^ These properties enable diverse applications in the fields of structural materials, optical detection, sensing, and energy harvesting.^9−15^ For instance, engineering-ordered diphenylalanine microrod arrays with uniform polarization demonstrated a significant piezoelectric constant of 17.9 pm V^–1^ that produced an open-circuit voltage of up to 1.4 V and achieved a power density of 3.3 nW cm^–2^ in nanogenerator devices.^16^ Moreover, encasing γ-glycine films in poly(vinyl alcohol) generated piezoelectricity and mechanical flexibility sufficient for *in vivo* sensing.^17^ However, achieving the control and optimization of their assembled structures and properties remains a significant challenge.

The distinctive properties of biomaterials, which enable their diverse applications, are closely related to the supramolecular packing mode.^18^ In general, the packing mode is influenced by the formation of hydrogen bond networks in terms of their dimensionality.^19^ For example, in two-dimensional (2D) layered structures, adjacent molecules within layers are bound by specific, directional noncovalent intermolecular interactions, such as dense hydrogen bonds or π–π interaction networks, which confer rigidity and mechanical strength.^20^ The binding between layers is typically weaker, being generally mediated by nonspecific van der Waals forces between hydrophobic side chains. This dimensional heterogeneity endows the materials with high flexibility, along with in-plane toughness, resulting in robust yet bendable, adaptable micro/nanostructures.^20^ In contrast, supramolecular architectures with 3D hydrogen bonding networks naturally exhibit higher mechanical strength and stability,^21^ which makes them suitable deformation-resistant components in electromechanical systems. Consequently, understanding and controlling the dimensionality of hydrogen bond networks in supramolecular arrangements is crucial and fundamental for the full potential of unlocking functional bioderived and bioinspired materials. While layered structures connected by 2D hydrogen bond networks are commonly observed in biological supramolecular architectures, including amino acid and short peptide assemblies, three-dimensional (3D) networks are rarer.^22−25^ Current studies on the modification of material dimensionality primarily focus on altering of their macroscopic morphologies through the selective use of growth templates, solvents, pH values, and other environmental factors.^15,26−30^ The potential to unlock diverse peptide material functions through modulating the dimensionality of hydrogen bond networks at the atomic level has often been underexplored. Such comprehension and control require a full understanding the relationship between molecular structures, lattice arrangements, and physical properties. Moreover, hydroxyl groups have been demonstrated to act as hydrogen bond donors, facilitating the formation of dense hydrogen bond networks.^22^ Inspired by this, we speculate that the addition of hydroxyl groups in short peptide molecules plays an important role in determining the hydrogen bond network dimensionality of supramolecular packing.

Here, we achieve two distinct packing structures of very similar cyclic dipeptides: a 2D water-mediated layered structure and a dry 3D network structure. This was realized through the rational design of functional groups within the engineered peptide structures (Figure 1). The simple insertion of a hydroxyl group provided an additional hydrogen bond donor that connects adjacent peptide molecules, triggering the formation of the zigzag-like 3D supramolecular stacking, as confirmed by X-ray crystallography. In the absence of the hydroxyl group, the molecule coordinated with water molecules to form 2D hydrogen bond layers that were stabilized by only weak dispersive van der Waals forces between diketopiperazine rings in the interlayer stacking. The 2D layered structure with in-plane β-sheet-like hydrogen bonds exhibited flexibility and a higher predicted piezoelectric response. In contrast, the hydroxyl-induced 3D hydrogen bond network demonstrated a mechanical stiffness that was 14-fold higher. Moreover, both assemblies demonstrated excellent thermostability, biocompatibility, and optical properties, making them suitable for imaging, sensing, and energy harvesting *in vivo*. Our work provides insight into the self-assembly of various dimensionalities of supramolecular packing simply by strategically optimizing the building blocks. By exploring the interplay between lattice arrangements and physical properties, we create new opportunities to generate nanomodified materials with micro and macroscopic mechanical, electrical, and optical properties tailored for diverse potential applications.^31,32^

**Figure 1:**
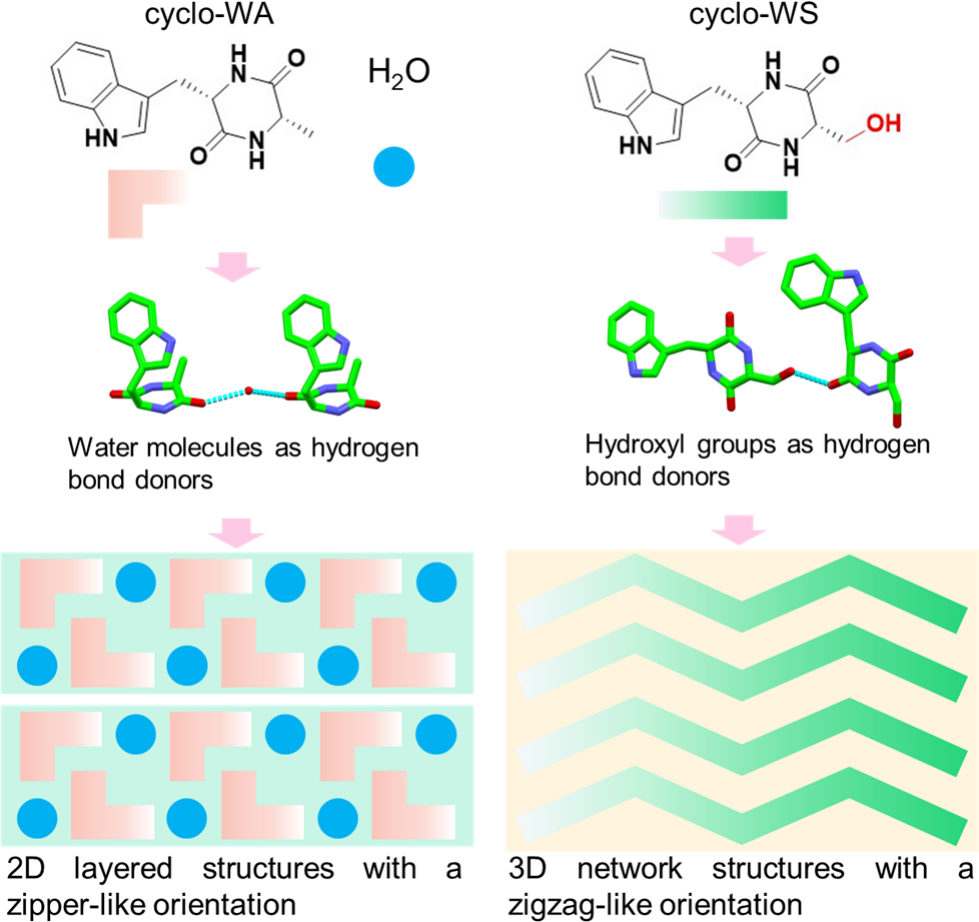
Schematic illustration of modulating the material dimensionality via hydroxyl-directed supramolecular packing of cyclo-dipeptide molecule building blocks.

We compared the assembly dimensionality of two types of cyclo-dipeptides, selected based on their anticipated supramolecular packing efficiencies determined by their hydrogen bond properties. Hence, we chose cyclo-tryptophan-alanine (cyclo-WA), which lacks hydroxyl groups, and cyclo-tryptophan-serine (cyclo-WS), which has a hydroxyl group on its hydroxymethyl side chain. The cyclo-WA molecules self-assembled into long prismoid-like structures, forming layer-by-layer stacking crystals in a water and methanol solution at room temperature (Figure 2a,b and Figure S1) or in water at −5 °C (Figure S2), as observed using scanning electron microscopy (SEM) and optical microscopy. cyclo-WA crystals obtained through the slow evaporation of a water and methanol mixed solution were larger, likely due to the lower nucleation rate and the availability of ample building blocks. Conversely, the cyclo-WA crystals formed by cooling in water exhibited smaller sizes, due to limited diffusion, which induced a low growth rate. Under the same growth condition, the cyclo-WS molecules self-assembled into distinctly different, half-moon-like structures with smooth surfaces in water at −5 °C (Figure 2c,d and Figure S3). This distinct morphology suggests varying supramolecular architectures resulting from the addition of a hydroxyl group to the molecular building block. The diverse supramolecular packing was confirmed through powder X-ray diffraction (XRD) measurements (Figure S4), revealing distinct diffraction peak positions that indicate variations in the crystal structures. Cyclo-WA crystals obtained in both water–methanol mixtures at room temperature and pure water at −5 °C exhibited the same structure (Figure S4), indicating the diversity of growth approaches. Powder XRD patterns for both cyclo-WA and cyclo-WS assemblies were highly consistent with the simulated XRD patterns from their single crystals (Figure 2e,f), confirming a high phase purity. Notably, the diffraction peaks at 6.7°, 13.5°, 20.2°, 27.0°, and 34.1° corresponded to the (001), (002), (003), (004), and (005) crystal planes of the cyclo-WA crystal, respectively (Figure 2e and table S1). Similarly, the diffraction peaks of the cyclo-WS crystal at 13.0°, 16.2°, 17.4°, 23.4°, and 26.2° corresponded to the (011), (110), (021), (012), and (022) crystal planes (Figure 2f and table S1), respectively, confirming the different supramolecular packing modes.

**Figure 2:**
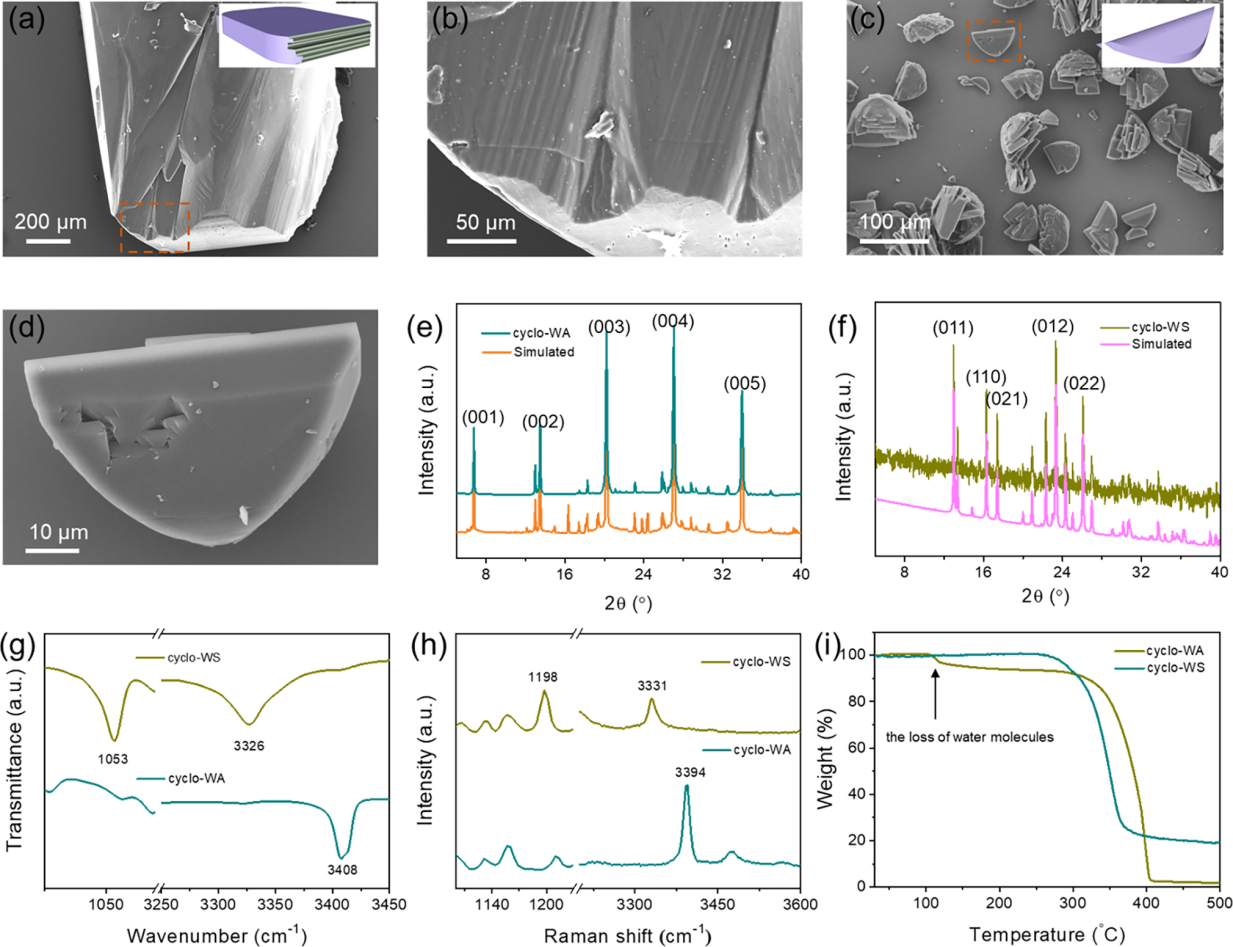
(a) SEM image of the cyclo-WA crystal obtained from a water and methanol solution, with the layer-by-layer stacking structure illustrated in the inset panel. (b) Zoomed-in view of the cyclo-WA crystal (marked with a red dotted rectangle in a). (c) SEM image of cyclo-WS crystals obtained from a water, with the smooth continuous 3D surface illustrated in the inset panel. (d) Zoomed-in view of the cyclo-WS crystal (marked with a red dotted rectangle in c). (e, f) XRD patterns of (e) cyclo-WA and (f) cyclo-WS crystals. (g) FTIR spectra of cyclo-WA and cyclo-WS crystals. (h) Raman spectra of cyclo-WA and cyclo-WS crystals. (i) Thermostability of cyclo-WA and cyclo-WS crystals.

Fourier transform infrared spectroscopy (FTIR) measurements confirmed the formation of different hydrogen bond types and patterns in the cyclo-WA and cyclo-WS assemblies (Figure 2g). In cyclo-WA crystals, a peak located at ∼3408 cm^–1^ indicated the presence of hydrogen-bonded water molecules,^33^ indicative of ordered crystallographic water molecules trapped in the crystal structure. No water bands were observed in cyclo-WS crystals, and instead, a peak at 3326 cm^–1^ corresponding to the peptide – OH band was detected,^34^ indicating the absence of ordered water molecules and the presence of the hydroxyl-functional group. A peak at 1053 cm^–1^ was attributed to the bending modes of C–OH groups coupled with the C–O stretching.^35^ In summary, cyclo-WA formed crystals through bridging with water molecules, whereas cyclo-WS provided an additional hydrogen bond donor through the hydroxyl-functional group to directly bind adjacent molecules, resulting in crystal formation. These findings were further supported by Raman measurements (Figure 2h). The peak at 3394 cm^–1^ was assigned to the H_2_O bending mode in cyclo-WA crystals, while the peaks at 1198 and 3331 cm^–1^ reflected the – OH stretching mode in cyclo-WS crystals.^36−38^

To assess the structural thermostability of the cyclo-WA and cyclo-WS crystals stabilized by the water-mediated and direct hydrogen bond modes, respectively (Figure 1), we performed thermal gravimetric analysis (TGA). As expected, the water molecules of cyclo-WA were lost at ∼101 °C, and the assembly started to degrade at ∼344 °C (Figure 2i). Cyclo-WS displayed similar high thermostability up to ∼308 °C (Figure 2i). The high thermal stability of the crystals may result from supramolecular architectures built from both hydrogen bonding and aromatic interactions.

Single-crystal XRD characterizations of as-prepared cyclo-WA and cyclo-WS crystals were carried out to provide insight into the two distinct hydrogen bond-mediated supramolecular packing modes. Cyclo-WA and cyclo-WS single crystals were obtained using the same growth condition to eliminate the influence of temperature and solvent on the assembly process. The structures revealed the atomic-scale features of the assemblies both in the absence of the hydroxyl group (cyclo-WA) and in its presence (cyclo-WS) within the peptide building unit. The crystal structure of cyclo-WA assemblies was determined to be a monoclinic space group *P*2_1_ with cell parameters of a = 6.2780 Å, b = 7.9007 Å, and c = 13.2593 Å (Table S1 and Figure S5). The asymmetric building unit consisted of one cyclo-WA and one water molecule (Figure 3a), while the unit cell contained two peptide molecules and two water molecules (Figure S6). Notably, the water molecule served as a hydrogen bond donor, facilitating connections between adjacent molecules (Figure 3b). In the crystallographic  direction, molecules packed into a β-strand-like arrangement that was stabilized by a pair of hydrogen bonds between oxygen and nitrogen atoms on adjacent diketopiperazine rings, with N(H)_backbone_... C=O (donor··· acceptor) distances of 3.009 and 3.004 Å, respectively (Figure 3c). The strands were connected through oxygen atoms that formed bonds with interstitial water molecules, with an O(H)_water_... C=O (donor··· acceptor) distance of 2.862 Å (Figure 3c), to form supramolecular β-sheets that further extended into a single layer with a 2D hydrogen bond network. In the crystallographic  direction, another parallel layer was stabilized by aromatic interactions of side-chain indole rings and packed in an interlocked zipper-like orientation, forming a double-layer structure (Figure 3d). The aromatic indole rings were arranged in an “edge-to-face” configuration, with a distance of 3.45 Å between the nearest aromatic indole rings (Figure S7). Weak van der Waals forces connected the layers (Figure 3d and Figure S8). Therefore, cyclo-WA, with its 2D hydrogen bond networks, allows layer-by-layer supramolecular stacking, resulting in long prismoid-like structures (Figure 2a). This layered structure differs from that of typical reported peptides, where interlayer molecules are stabilized primarily by aromatic interactions, without the formation of 2D hydrogen bond networks.^20,39^

**Figure 3:**
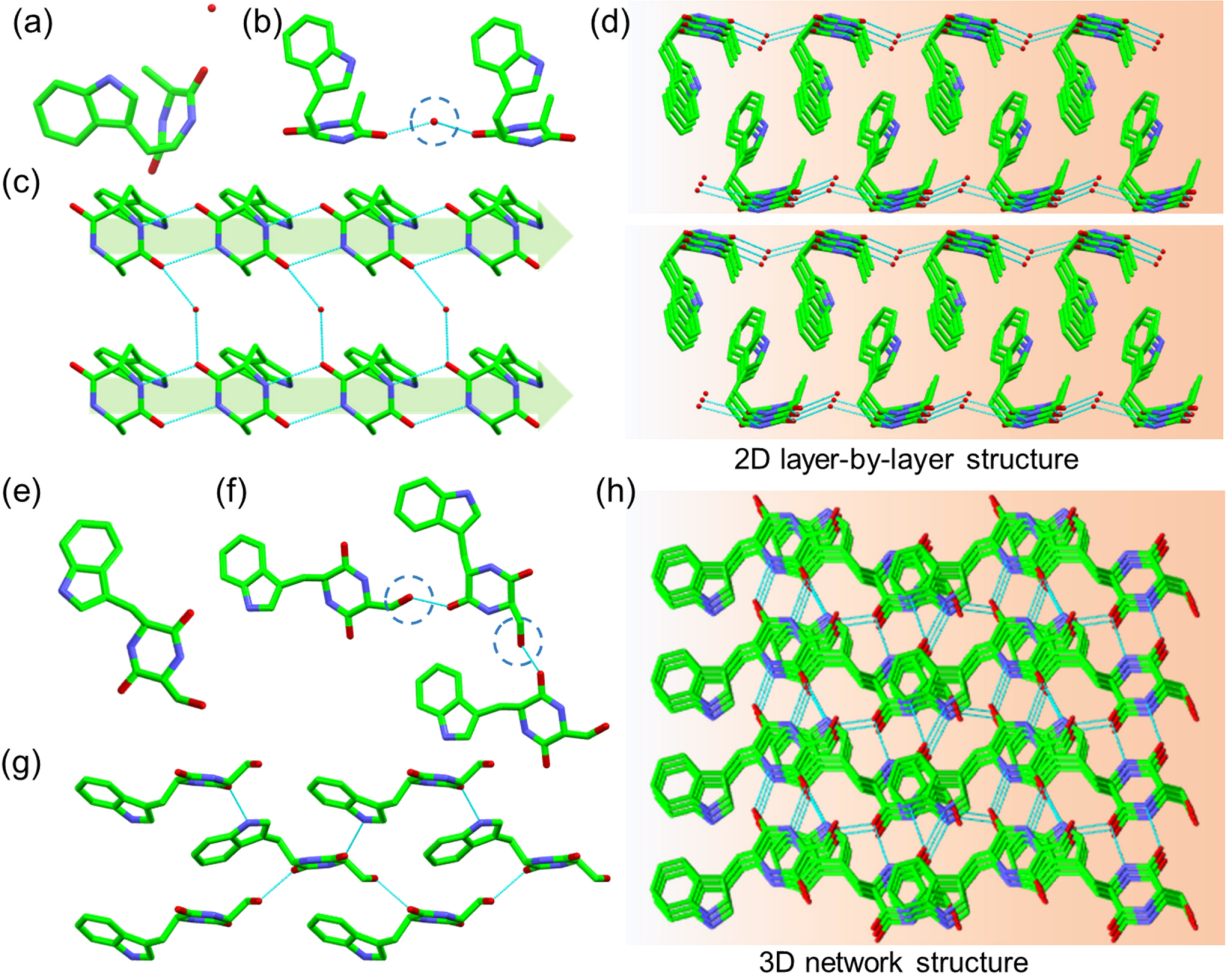
(a) The cyclo-WA molecular asymmetric unit with an ordered water molecule. Color code: green, C; blue, N; red, O, with hydrogens omitted for clarity. (b) Water molecules acting as hydrogen bond donors to bridge the cyclo-WA molecules. (c) The single-layer arrangement of cyclo-WA molecules. (d) The cyclo-WA molecules packing into zipper-like units and creating a 2D layer-by-layer structure. (e) The cyclo-WS molecular asymmetric unit. (f) Hydroxyl groups acting as hydrogen bond donors to connect the cyclo-WS molecules. (g) The cyclo-WS molecules connected in dense hydrogen bond networks. (h) The cyclo-WS molecular arrangement in a 3D zigzag-like network.

Furthermore, the cyclo-WS crystal shared the same space group (*P*2_1_) as the cyclo-WA crystal but had different cell parameters (a = 6.1773 Å, b = 13.3209 Å, and c = 8.1672 Å) (Table S1 and Figure S9). The asymmetric building unit comprised a single peptide molecule (Figure 3e), and two peptide molecules were contained in a unit cell (Figure S10) without water molecules. The serine hydroxyl group served as a hydrogen bond donor, directly connecting adjacent molecules without the need for intermediate water molecules (Figure 3f). In the *bc* plane, one cyclo-WS molecule was linked to four adjacent molecules by forming two pairs of hydrogen bonds (Figure 3g). The indole rings were bound to diketopiperazine rings on the neighboring molecules, with an N(H) ... C=O (donor··· acceptor) distance of 2.873 Å. In the backbone, one oxygen atom connected to the hydroxyl group on the adjacent molecule, and another oxygen atom interacted with the nitrogen atom on the next indole ring. An additional hydrogen bond was formed between the hydroxyl group and a diketopiperazine ring on an adjacent molecule, with an O(H)_hydroxyl_... C=O (donor··· acceptor) distance of 2.729 Å. These molecules aligned along the *a*-axis and packed in a zigzag-like orientation in the crystallographic  direction, creating a 3D hydrogen bond network (Figure 3h). The addition of a hydroxyl group triggered a dramatic transformation in the supramolecular packing, transitioning from a 2D layered to a 3D network structure, by providing a hydrogen bond donor to directly connect adjacent molecules. Therefore, increasing the number of hydrogen bond sites, such as hydroxyl groups, in molecules promotes the formation of architectures with 3D hydrogen bond networks. With fewer of these sites, molecules tend to form crystal structures with 2D or one-dimensional (1D) hydrogen bond networks.

The diverse dimensionality of supramolecular packing reflects the difference in hydrogen bond interactions, which can be investigated by Hirshfeld surface analysis, a useful tool for predicting the intermolecular interactions in organic crystals.^40−42^ In the cyclo-WA *d*_norm_ surface, areas denoted in red indicated strong O···H and H···O interactions, resulting from the formation of hydrogen bonds between dipeptide and water molecules (Figure S11a). In contrast, the red regions in the cyclo-WS *d*_norm_ surface were not only located near indole and diketopiperazine rings but also in proximity to the hydroxyl group, which serves as an additional hydrogen bond donor (Figure S11b). In addition, the blue area highlighted less polar interactions, including aromatic interactions and van der Waals forces. The detailed contribution of the interactions between O and H atoms to overall interatomic interactions was quantified in 2D fingerprint plots. As shown in Figure S11c and S11d, cyclo-WS, with its 3D networks, exhibited a higher contact between H and O atoms compared to cyclo-WA with 2D layers, suggesting stronger hydrogen bond strength in cyclo-WS.

The hydrogen bond modes and overall strength of the supramolecular packing affect the mechanical strength of assemblies, which can be evaluated by Young’s modulus and point stiffness measurements.^43,44^ Using atomic force microscopy (AFM), the tip was scanned over the surface of cyclo-WA and cyclo-WS single crystals with a smooth area of 5 × 5 μm^2^. We determined Young’s modulus values of cyclo-WA and cyclo-WS crystals (Figure 4a-f and Figure S12–S14) by fitting force–displacement traces collected at different points. The measured planes were the (001) crystal plane for cyclo-WA and cyclo-WS, corresponding to the largest predicted plane, according to the Bravais, Friedel, Donnay, and Harker (BFDH) theory (Figure S15).^45,46^ The cyclo-WA crystals displayed a relatively low Young’s modulus of ∼3.5 GPa, suggesting mechanical flexibility (Figure 4b). On the other hand, the Young’s modulus of cyclo-WS crystals was much higher at approximately ∼49 GPa (Figure 4e), which is about 14 times greater than that of cyclo-WA. A significant enhancement in the point stiffness was observed from 56.6 N m^–1^ for cyclo-WA to 407.9 N m^–1^ for cyclo-WS (Figure 4c,f), indicating a 7× improvement in point stiffness. The dramatic improvement in the mechanical properties can be ascribed to the different packing architectures. The cyclo-WA molecules, connected by 2D hydrogen bond networks, exhibited weaker atomic interactions between layers, while the cyclo-WS molecules, linked by a 3D hydrogen bond network, demonstrated higher mechanical strength.^20,21^ Our approach demonstrates an efficient method to control the mechanical properties of biomaterials by engineering the building unit through a single site substitution of H → OH to create the desired 3D hydrogen bond network.

**Figure 4:**
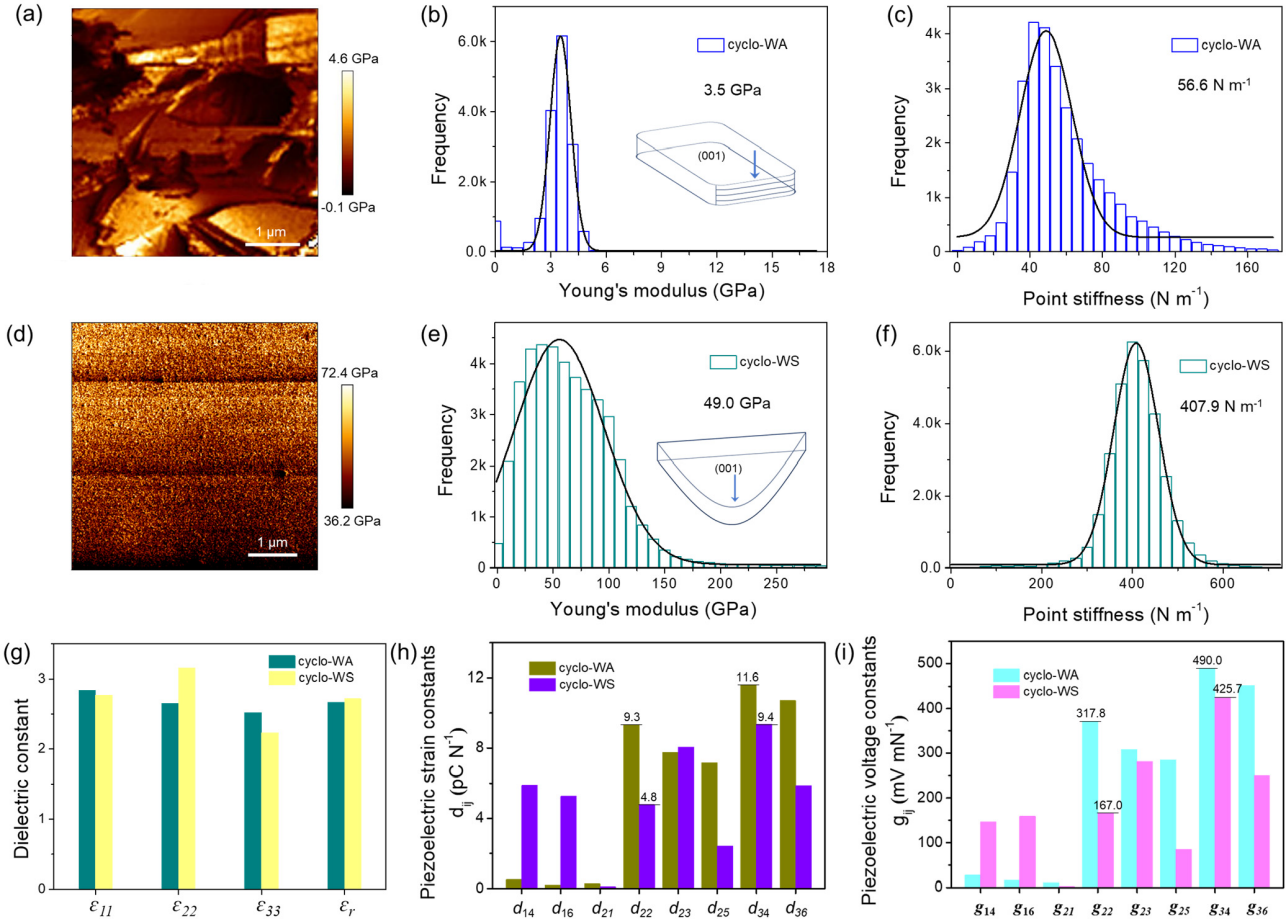
(a-f) Comparison of (a-c) cyclo-WA and (d-f) cyclo-WS. (a, d) Topographic modulus maps. (b, e) The statistical Young’s modulus distributions. (c, f) Statistical point stiffness distributions. (g-i) DFT-predicted (g) dielectric constants where ε_r_ represents the average dielectric constant, (h) piezoelectric strain constants and (i) voltage constants for cyclo-WA, and cyclo-WS crystals.

The noncentrosymmetric structures of cyclo-WA and cyclo-WS crystals indicate that they will exhibit some degree of electromechanical response. The size of the piezoelectric response of biomaterials, namely the generation of voltage in response to deformation, is associated with the directionally aligned hydrogen bonds generating net uncompensated supramolecular dipoles.^47^ The piezoelectric charge constants, piezoelectric strain constants, and piezoelectric voltage constants, as well as the dielectric constants of cyclo-WA and cyclo-WS crystals, were predicted by density functional theory (DFT) calculations (Figure 4g-i and Table S2,S3). The cyclo-WA and cyclo-WS crystals have the same space group, *P*2_1_, resulting in a similar-shaped piezoelectric tensor, including three out-of-plane and five shear nonzero piezoelectric coefficients. Cyclo-WA exhibited significantly higher out-of-plane and shear piezoelectric charge constants compared to cyclo-WS. Especially, the cyclo-WA crystals exhibited the highest out-of-plane and shear piezoelectric coefficients of d_22_ = 9.3 pC N^1–^, and d_34_ = 11.6 pC N^1–^, respectively (Figure 4h). In contrast, the cyclo-WS crystals showed smaller piezoelectric responses with an out-of-plane and a shear piezoelectric coefficient of d_22_ = 4.8 pC N^1–^, and d_34_ = 9.4 pC N^1–^, respectively (Figure 4h). The larger response for cyclo-WA is consistent with the softer layered structure, which facilitates the piezoelectric polarization under the same applied force. The continuous 3D hydrogen bond network observed for cyclo-WS results in high stiffness along the longitudinal direction (Figure 4e,f), accompanied by lower out-of-plane piezoelectricity. Furthermore, a calculated maximum piezoelectric voltage constant (*V*_max_) value on the order of 0.5 V (Figure 4i) suggests potential applications for the cyclo-dipeptides in implanted actuators, strain sensors, and energy harvesting devices, with predicted g_34_ of 490.0 mV mN^–1^ and 425.4 mV mN^–1^ for cyclo-WA and cyclo-WS, respectively.

To test the potential suitability of the peptides as implanted biomaterials, we investigated the biocompatibility of cyclo-WA and cyclo-WS crystals toward HeLa cells by an MTT-based cell viability assay. This was necessary as the utilization of organic solvents during materials synthesis and crystal growth may reduce the biocompatibility of both natural and bioinspired peptides, which in turn limits the application of peptides as implantable materials in the fields of biomedicine, bioengineering, and energy harvesting *in vivo*.^48^ More specifically, it is essential to determine the influence of functional groups, including the – OH site, on the cellular toxicity of the peptides. As displayed in Figure S16, both as-prepared cyclo-dipeptide assemblies showed high biocompatibility, in which the cellular viability of cells grown in the presence of 1 mg mL^–1^ cyclo-WA and cyclo-WS reached 80% and 82%, respectively after incubating at 37 °C for 24 h (Figure S16). Although the increase in peptide concentration decreased cellular viability, it was still maintained at near 60% for cyclo-WA and more than 60% for cyclo-WS at a high concentration of 10 mg mL^–1^ (Figure S16). Following the good cell viability (negligible cytotoxicity or no toxicity at all) assays, we examined the *in vitro* interaction of human cells with cyclo-WA and cyclo-WS crystals after 24 h (Figure S17). We observed very light green fluorescence residing in the cytoplasm (Figure S17), and very high fluorescence in the nucleus (after staining with life cell nucleus dye (Hoechst dye) in the nucleus). Moreover, we noticed that the cell is healthy even after treatment with cyclo-WA and cyclo-WS crystals, which suggests that the above-synthesized crystal is highly biocompatible. The result was further confirmed by the live/dead analysis of cells using live-cell confocal microscopy (Figure S17), indicating the excellent biocompatibility of cyclo-WA and cyclo-WS crystals. For future applications, it would be interesting to evaluate cell viability over a longer time postincubation with cyclo-peptides, extending beyond the standard 24-h live/dead assay used in the current work. Combining the high predicted piezoelectric response (Figure 4h,i) with the measured biocompatibility, the dipeptide crystals show significant potential for implanted electronic device applications.

Protons can move along hydrogen bonds and water molecules in crystals, lowering the conductance band gap between valence-occupied levels and unoccupied electronic states.^49^ To study the electronic properties of the two distinct assemblies, we characterized the electronic band structures and density of states (DOS) properties of cyclo-WA and cyclo-WS crystals using DFT calculations. The cyclo-WA structure with bridging water molecules showed a band gap of 3.21 eV above a Fermi level at 1.21 eV (Figure 5a,b). Cyclo-WS showed a slightly larger band gap (3.45 eV) (Figure S18), which may reflect the absence of water-based proton hopping sites in cyclo-WS.^49^ The calculated band gaps of cyclo-WA and cyclo-WS of 3.21 and 3.45 eV, respectively, are consistent with the trend and close to systematic slight underestimation^50^ to the measured values (3.33 eV for cyclo-WA and 3.87 eV for cyclo-WS) obtained from UV–visible absorption spectroscopy (Figure S19). The corresponding wavelengths for cyclo-WA and cyclo-WS are 375 and 366 nm, respectively.

**Figure 5:**
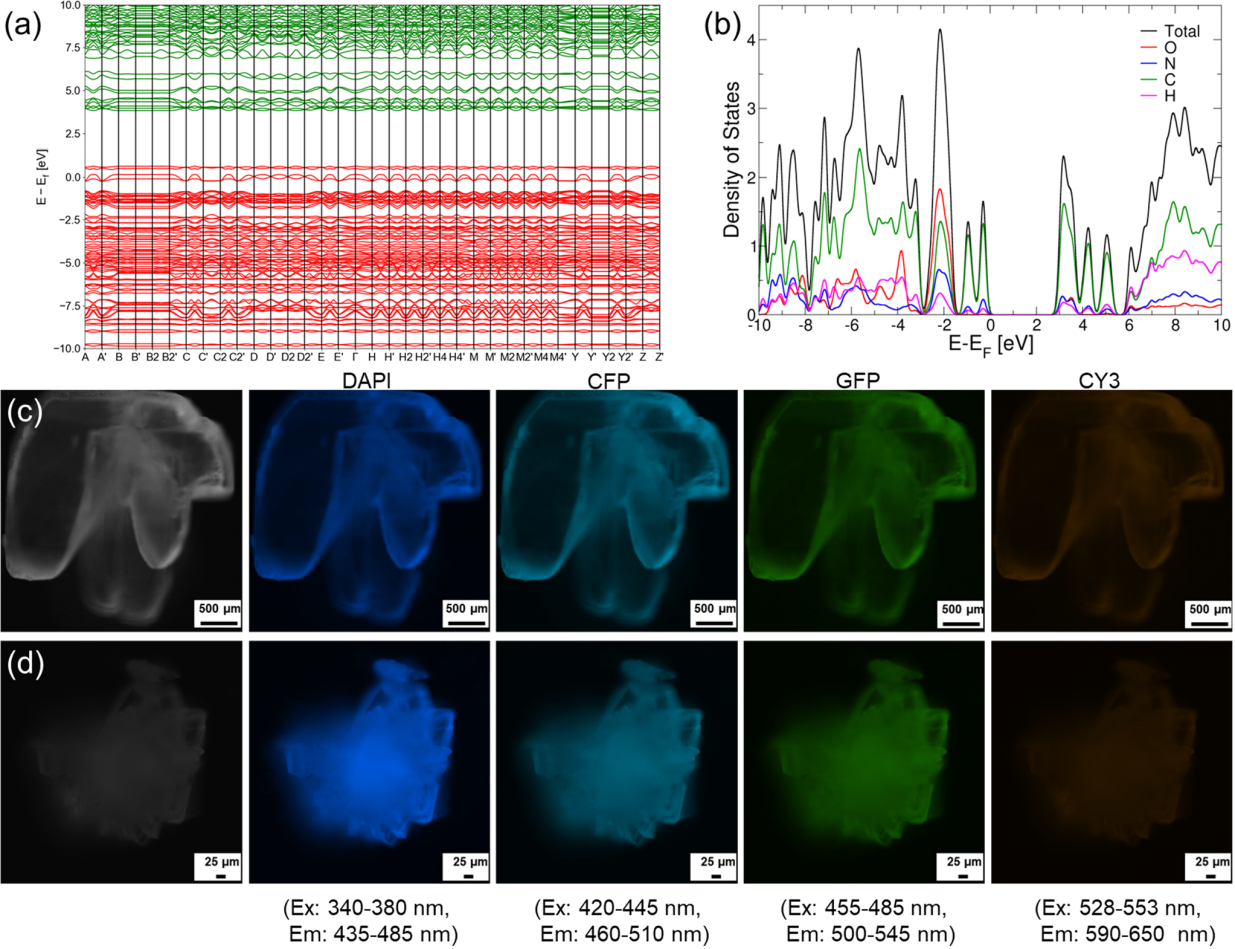
(a) Calculated band structure of the cyclo-WA crystal. (b) The corresponding projected-to-atoms density of states with the Fermi energy level shifted to zero. The calculated electronic properties of cyclo-WS are shown in Figure S18. (c, d) Microscopy images of (c) cyclo-WA and (d) cyclo-WS crystals in a dark field and in different fluorescence channels, as indicated.

The proton transfer between hydrogen bond donors and acceptors also results in lower electron excitation energies, thereby improving fluorescence.^51^ Furthermore, supramolecular structures containing aromatic residues can potentially exhibit high fluorescence properties, as the dipolar coupling between the excited states of aromatic residues contributes to fluorescence.^50^ Based on our measurements (Figure 5c,d), both cyclo-WA and cyclo-WS assemblies emitted blue, cyan, green, and weak yellow fluorescence under UV light excitation. These fluorescence properties may arise from a combination of aromatic interactions, hydrogen bonds, and a red-edge excitation shift.^52^ However, the assemblies showed variable degrees of fluorescence, with the cyclo-WS assembly effectively invisible by yellow fluorescence (Figure 5d). Fluorescence emission spectra were recorded for cyclo-WA and cyclo-WS assemblies to verify their fluorescent properties. Cyclo-WA crystals emit at approximately 332 and 387 nm in the UV region when excited between 280 and 310 nm and 320–350 nm, respectively (Figure S20a). Additionally, these crystals exhibit blue light emission when excited between 360 and 410 nm (Figure S20a). Conversely, cyclo-WS crystals show emission at about 328 nm with excitation from 280 to 300 nm (Figure S20b). Notably, the emission shifts from the UV to the blue light region as the excitation wavelength increases from 310 to 410 nm (Figure S20b).

In this work, we demonstrated two different dimensionalities of supramolecular packing of cyclo-dipeptides: 2D layered and 3D network structures with distinct macroscale properties, achieved simply by replacing one hydrogen atom with a hydroxyl group to insert a key hydrogen bond moiety. The cyclo-dipeptide molecule lacking hydroxyl groups formed 2D layered structures through connected water-mediated hydrogen bonds, stabilized by zipper-like motifs and weak van der Waals contacts between the layers. In contrast, the addition of a hydroxyl group on the molecule provided a hydrogen bond donor to directly connect adjacent molecules in a water-free zigzag-like 3D hydrogen bond network. As a result, the dimensional, optical, mechanical, piezoelectric, and electronic characteristics can be modulated. Specifically, the 3D network structure demonstrated a 14-fold increase in stiffness compared to the 2D structure. Meanwhile, the water-mediated 2D layered structure, with weak interlayer interactions exhibited a smaller predicted band gap and higher predicted piezoelectricity along the longitudinal direction, whereas the 3D network structure generating a chemically and physically more rigid structure with weaker electromechanical response overall. Moreover, an MTT-based cell viability assay confirmed the good biocompatibility of both cyclo-dipeptide crystals, underscoring their potential for *in vivo* applications. Our findings provide a base for understanding how diverse supramolecular packing modes embed functionality in 2D layered and 3D network structures, paving the way to programmable dimensionality of hydrogen bond networks at the atomic level and thus programmable properties for a wide range of self-assembled materials.

## Supporting Information Available

Experiment method, morphology, structure, van der Waals forces, Hirshfield surfaces, and property characterizations including SEM images, single-crystal structural analysis, powder XRD, mechanical properties, DFT calculated results, mechanical, electrical, piezoelectric, and biocompatible property of cyclo-WA and cyclo-WS crystals (PDF)
Crystallographic data (CCDC 2304390) (CIF)
Crystallographic data (CCDC 2304391) (CIF)

## Supplementary Material

tz4c00665_si_001.pdf

tz4c00665_si_002.cif

tz4c00665_si_003.cif
