# Supplementary material for: The Dimensionality of Hydrogen Bond Networks Induces Diverse Physical Properties of Peptide Crystals
Source: ACS Mater Lett. 2024 Jul 23;6(8):3824–33. doi: 10.1021/acsmaterialslett.4c00665 (PMC11304472; doi:10.1021/acsmaterialslett.4c00665)
Supplement: Supplementary file 1 — tz4c00665_si_001.pdf [file tz4c00665_si_001.pdf]

Supporting Information

The Dimensionality of Hydrogen Bond Networks Induces Diverse Physical Properties of Peptide Crystals

Hui Yuan,[a] Pierre-Andre Cazade,[b] Chengqian Yuan,[c] Bin Xue,[d] Vijay Bhooshan Kumar,[a] Rusen Yang,[e] Gal Finkelstein-Zuta,[a] Lihi Gershon,[a] Maoz Lahav,[a] Sigal Rencus-Lazar,[a] Yi Cao,[d] Davide Levy,[f] Damien Thompson,[b,*] Ehud Gazit [a,*]

[a] The Shmunis School of Biomedicine and Cancer Research, George S. Wise Faculty of Life Sciences; Tel Aviv University, Tel Aviv 6997801, Israel

[b] Department of Physics, Bernal Institute, University of Limerick, Limerick, V94 T9PX, Ireland

[c] State Key Laboratory of Biochemical Engineering, Institute of Process Engineering, Chinese Academy of Sciences, Beijing, China

[d] National Laboratory of State Microstructure, Department of Physics, Nanjing University, Nanjing, 210093, Jiangsu, China.

[e] Academy of Advanced Interdisciplinary Research, School of Advanced Materials and Nanotechnology, Xidian University, Xi'an 710126, China

[f] Center for Nanoscience and Nanotechnology, Wolfson Applied Materials Research Center, University of Tel Aviv, Tel Aviv 6997801, Israel

E-mail: ehudg@post.tau.ac.il (E. G.); damien.thompson@ul.ie (D. T.)

EXPERIMENTALSECTION

Crystal growth: Cyclo-WA assemblies were obtained through two different methods. For the first method, 12 mg cyclo-WA was dissolved in a 4:1 methanol-water mixture at the concentration of 2 mg mL^{-1}, achieved by heating at 70 ℃. 4 mL of the solution was transferred into a glass vial, and a Parafilm with six holes was sealed over it. The crystals were left to incubate at ambient conditions and, after four weeks, grew into long prismoid-like structures. The second method involved dissolving 5 mg of cyclo-WA powder in 2 mL of water by heating at 90 ℃ for 1 h. The transparent solution was obtained by filtration using filter paper and then incubated at -5 ℃. Long prismoid-like assemblies formed after three weeks. For Cyclo-WS, the powder was dissolved in water at a concentration of 10 mg L^{-1} in a glass vial by heating at 70 ℃ until the solution became transparent. The system was then incubated at -5 ℃. After two weeks, the peptide crystallized into half-moon-like structures. In both cases, crystals were collected by filtration and vacuum drying at room temperature.

Scanning electron microscopy: Crystal solutions at the concentration of 1 mg mL^{-1} were uniformly distributed on silicon substrates and dried at room temperature by a slow evaporation process. To enhance the conductivity, an Au film was sputtered on the samples. A JCM-6000PLUS Neo Scope Benchtop scanning electron microscope was employed for imaging.

Powder X-ray diffraction and thermogravimetric analysis: The dry cyclo-WA and cyclo-WS crystals were filled into a quartz zero-background sample holder. A D8 DISCOVER diffractometer was used to collect powder diffraction data with Cu Kα radiation and a scanning angle ranging from 5 to 40°. The thermostability of cyclo-WA and cyclo-WS crystals was characterized using a STA+449F5 simultaneous thermal analyzer, and the data was recorded at a heating rate of 10 ℃ min^{-1} in the Ar atmosphere.

Processing and structural refinement of crystal data: The cyclo-WA and the cyclo-WS single crystals were obtained through the same cooling process in supersaturated water solution and solvent as outlined above for the cyclo-WS crystals. The prepared crystals were covered with Par atone oil (Hampton Research), fixed in a Mateen cryo-loop, and rapidly frozen in liquid nitrogen. A Bruker Kappa Apex Duo single diffractometer with Cu Kα radiation was used for the cyclo-WA crystal data collection at 110 K. The cyclo-WS data was collected by Rigaku Synergy S equipped with a Pilatus Decries 300K Cttee detector with Mo Kα radiation at 113.15 K.

The Rigaku CrysAlis^{Pro} software was used to process the diffraction data. The crystal structures were solved and refined using Bruker SHELXTL software. Except for hydrogen atoms, all atoms were placed in calculated positions and refined in a riding mode. Parameters for data collection and refinement are provided in Table S1, and the final CIF files are given in the Supporting Information. The crystallographic data has been deposited in the CCDC with numbers 2304390 and 2304391.

Young's modulus and point stiffness measurements: Atomic force microscopy (AFM) nanoindentation was used to measure the Young's modulus of the crystals. All the experiments were performed using a commercial AFM (JPK, Nanowizard IV, Berlin, Germany). The crystals were spread on mica substrates and the cantilever was moved to the surface of the crystals. Nanoindentation was performed on the crystal surface (scan area: 5 μm × 5 μm) in QI mode (conditions: pixels: 60 × 60; Z length: 0.1 μm; extend and retract speed: 30 μm s^{-1}; Z resolution: 80000 Hz; maximum loading force: 1000 nN) and RTESPA-525 cantilevers (Bruker Company, half-open angle of the pyramidal face of θ: < 10°, tip radius: ~10 nm, spring constant: ~200 N m^{-1}) were used in all the experiments. Typically, the cantilever was extended to the surface of the crystal and retracted while indentation depths pressed by the cantilever tip were less than 8 nm. The force and displacement during the process were recorded.

The Young's modulus of the crystals was calculated by fitting the retraction curve with the Hertz model (1):

$$F=\frac{4}{3}\frac{E}{(1-\nu^2)}\sqrt{R}\delta^{3/2} \qquad (1)$$

F corresponds to the force, *δ* corresponds to the depth of the crystal pressed by the cantilever tip, *R* is the radius of the tip, *E* is the Young's modulus of the crystals, and ν is the Poisson ratio (ν = 0.3). The point stiffness was determined as the normal force divided by the deformation of the sample and calculated from the force-displacement curves after deducting the deformation of the cantilever. For each sample, at least six regions were randomly selected to perform the nanoindentation, and at least three cantilevers were used in the experiments to exclude a tip dependency of the results. All the data was analysed and the two-dimensional diagrams were reconstructed using the JPK data processing 7.0.46 software (JPK company).

DFT calculations: All modelling was performed using the CP2K package[1] for periodic density functional theory (DFT) calculations.[2] The Orbital Transformation[3] (OT) SCF algorithm was used with Goedecker, Teter and Hutter (GTH) type pseudopotentials and a molecular optimized double-zeta gaussian basis set (akin to 6–31G**).[4] The cut-off for the plane waves and gaussians was 900 Ry and 60 Ry, respectively, and energy was converged to 10^{-8} hartree. Exchange-correlation effects were treated using the Perdew, Burke, and Ernzerhof (PBE)[5]

implementation of the Generalised Gradient Approximation (GGA).[6] Grimme D3 dispersion corrections were used to capture van der Waals interactions. [7,8] All crystal structures were optimized using conjugate gradient minimization in a supercell model of 3 × 3 × 2 for cyclo-WA and 3 × 2 × 3 for cyclo-WS.[9] The piezoelectric parameters were calculated using a finite difference method, with the supercell strained by ± 0.015 in each of the Voigt directions. The piezoelectric tensor is then the response of polarization (considered here as a periodically corrected Berry phase) to the applied strain. The matrix product between the piezoelectric charge tensor, e, provides the piezoelectric strain tensor, *d*. The DOS distribution was obtained from the optimized supercell structure using a standard diagonalization and Γ-point sampling. The band structure, which requires large memory and computing time, was performed on a single optimized crystal unit cell using a standard diagonalization and multiple k-points based on the Monkhorst-Pack scheme: 3 × 3 × 2 for cyclo-WA, and 3 × 2 × 3 for cyclo-WS. Special k-points for the band structure path were generated with SeeK-path tools.[10] A sampling of 25 data points between consecutive special k-points was used.

Cell viability measurement: A total of $1x10^6$ HeLa cells mL^{-1} were cultured in 96-well tissue microplates (100 µl per well) and allowed to adhere overnight at 37°C for the analysis of cell viability. A fine powder of cyclo-WA or cyclo-WS crystals suspension was added to the cell growth medium at a concentration of 0.5, 1, 2, 5, and 10 mg ml^{-1}. The cells were seeded in one half of each plate, while the other half served as a blank control. A medium without cyclo-WA or cyclo-WS crystals was used as a negative control. To assess cell viability, 3-(4,5-dimethylthiazolyl-2)-2, 5-diphenyltetrazolium bromide was used according to the manufacturer's instructions after a 24-hour incubation at 37°C. Then 10 µl of the 5 mg/mL MTT reagent dissolved in PBS was added to each of the 96 wells, followed by another 3 h of incubation at 37°C. The wells were then filled with 100 µl of extraction buffer (100% DMSO) and incubated for 30 minutes at 37 °C in the dark. Finally, absorbance intensity was measured using a multi-plate reader at 570 nm, with background subtraction at 680 nm.

Live cell imaging: Confocal microscopy was used to obtain images of live HeLa cells grown in the presence of cyclo-WA or cyclo-WS crystals. Briefly, the cells were grown in glass bottom dishes to a confluence of 75%. Afterward, the cells were cultured in media containing cyclo-WA or cyclo-WS crystals at a concentration of 2 mg/mL for varying periods of time. Then, the cells were washed twice with PBS. Images were acquired using a Leica SP8 inverted confocal microscopy (Leica Microsystems, Wetzlar, Germany). The ranges of excitation and emission were as follows: for cyclo-WA/cyclo-WS crystals, λ_{ext} = 490 nm, λ_{em} = 510-590 nm; and for Hoechst live cell nucleus staining dye, λ_{ext} = 405 nm, λ_{em} = 420−500 nm. An additional barrier

filter was used to block emission light above 590 nm. It was separated by a dichroic mirror (555 nm) and the two fluorescent lights were filtered by two bandpass filters (500-550 nm and 540-690 nm).

SUPPLEMENTARY FIGURES

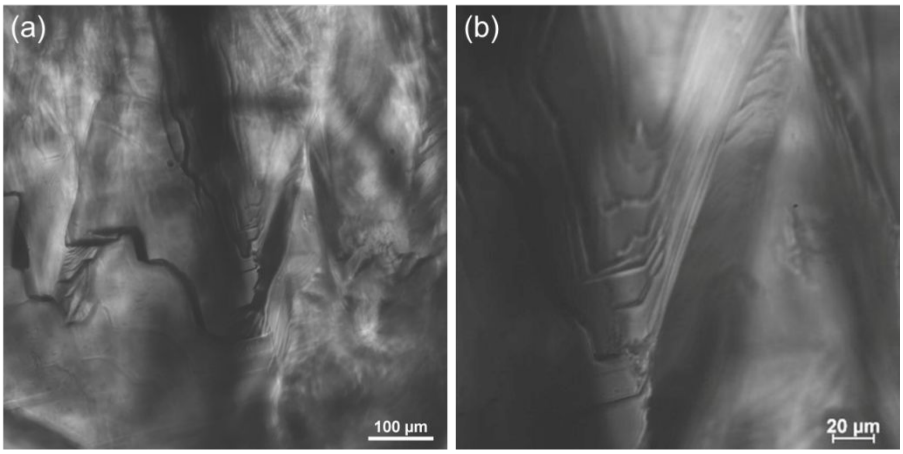

Figure S1 Microscopy images of cyclo-WA crystals obtained in a water and methanol mixed solution at room temperature.

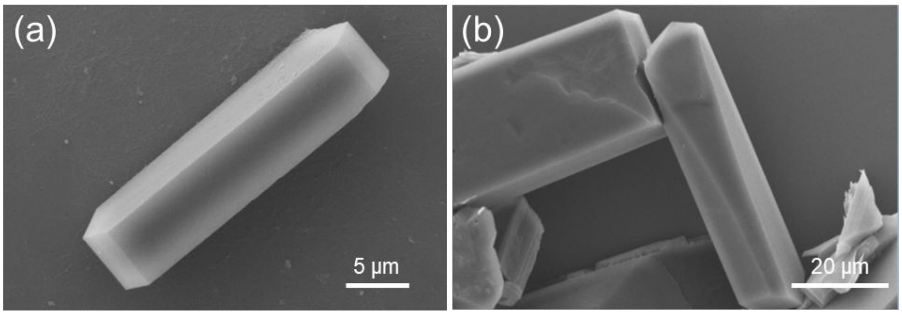

Figure S2 SEM images of cyclo-WA crystals obtained in water at -5 ℃.

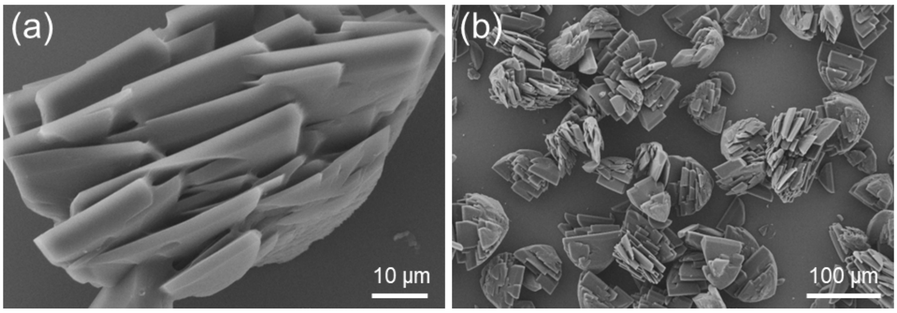

Figure S3 SEM images of cyclo-WS crystals obtained in water at -5 ℃. The cyclo-WS assembles into polycrystal structures where many crystals are stacked together. This may be induced by heterogeneous nucleation and an uneven distribution of temperature.

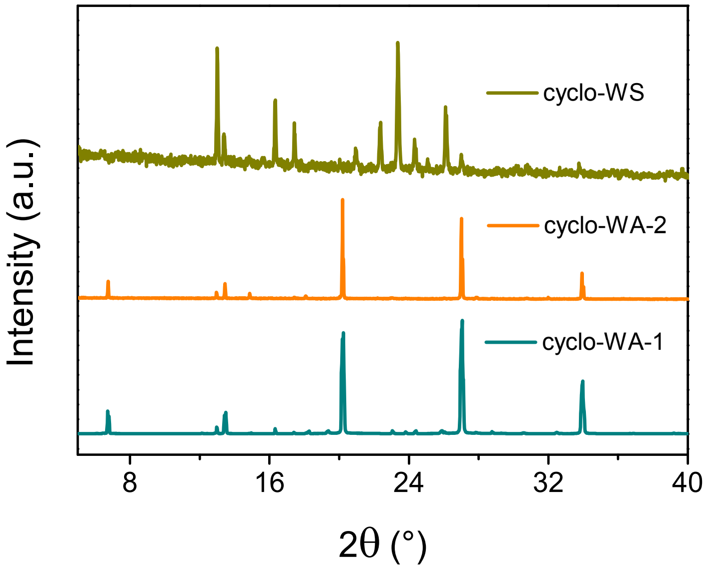

Figure S4 XRD patterns of cyclo-WA and cyclo-WS crystals. The cyclo-WA-1 crystal, obtained in water-methanol mixtures at room temperature, and the cyclo-WA-2 crystal, obtained in water alone at -5 ℃, both exhibit the same structure.

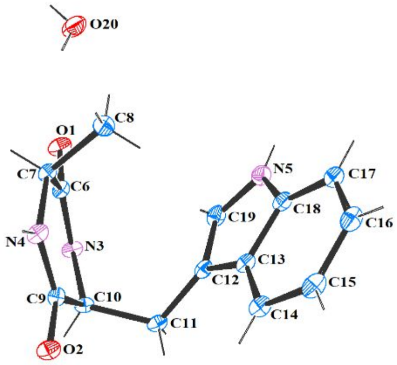

Figure S5 ORTEP diagram of the cyclo-WA crystal with ellipsoid probability of 50%.

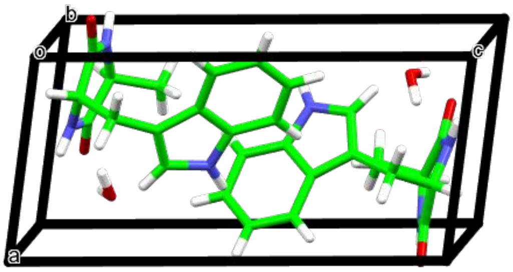

Figure S6 The unit cell of cyclo-WA crystals.

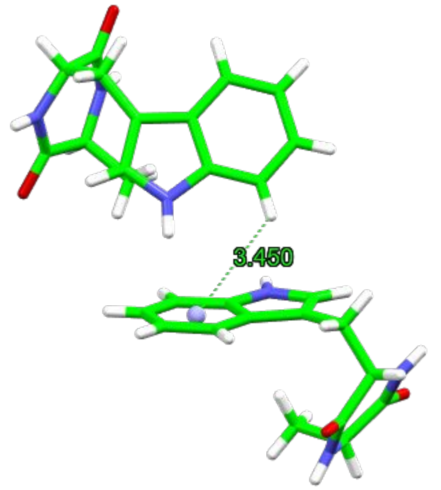

Figure S7 The “edge-to-face” interaction of cyclo-WA crystals.

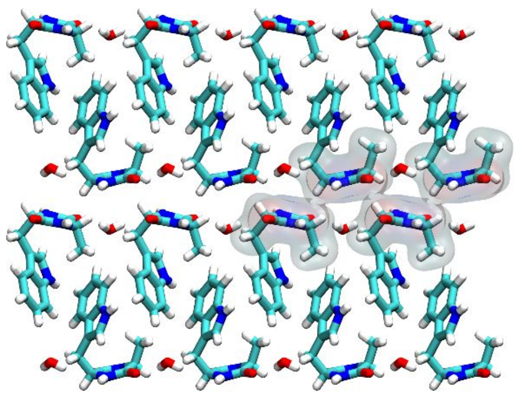

Figure S8 Overlay of space-filling spheres showing the weak van der Waals interactions mediating the layer-layer contacts. Color code: cyan, C; blue, N; and red, O.

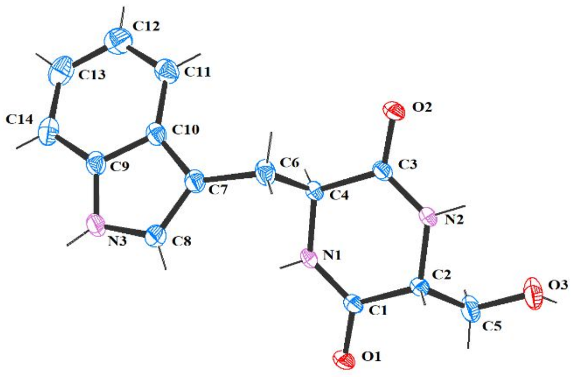

Figure S9 ORTEP diagram of the cyclo-WS crystal with ellipsoid probability of 50%.

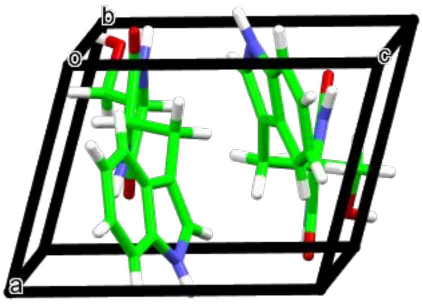

Figure S10 The unit cell of cyclo-WS crystals.

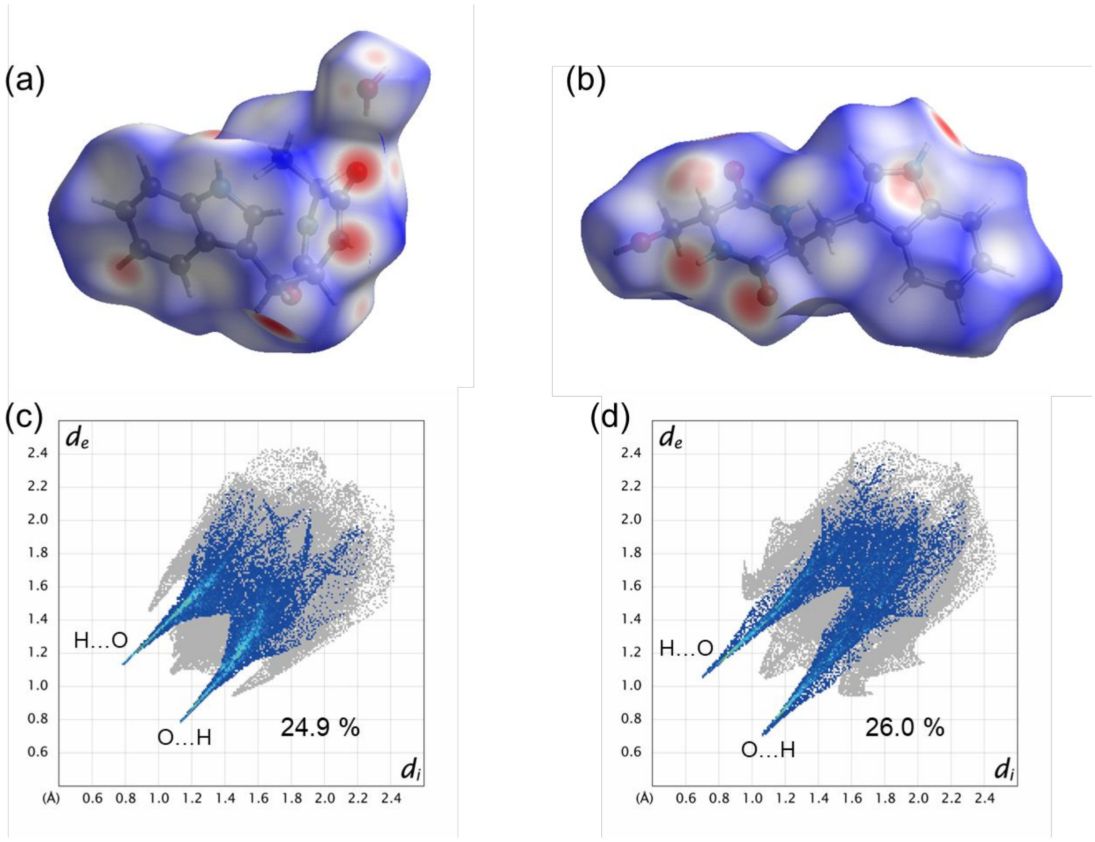

Figure S11 (a,b) Hirshfeld surfaces of (a) cyclo-WA, and (b) cyclo-WS mapped with d_{norm}. (c,d) 2D fingerprint plots of (c) cyclo-WA, and (d) cyclo-WS.

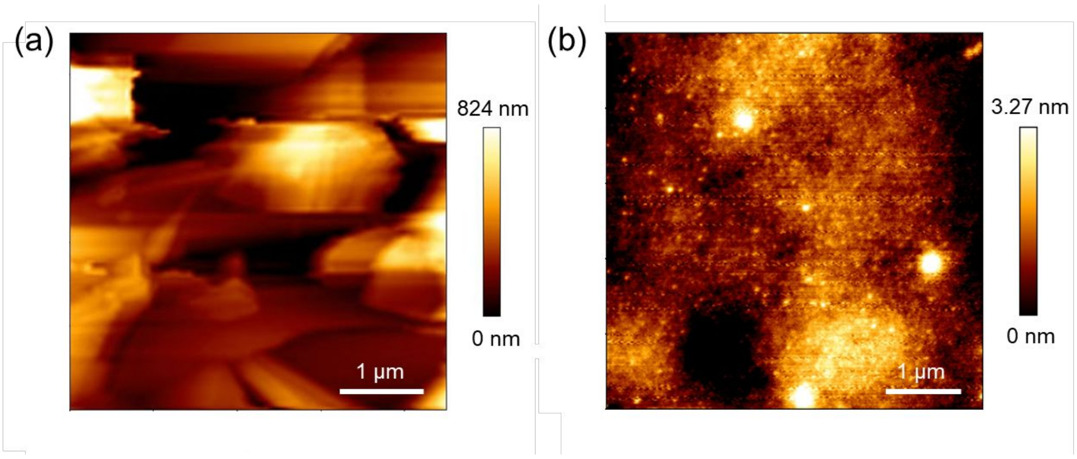

Figure S12 AFM topography images of (a) cyclo-WA and (b) cyclo-WS crystals. The significant heterogeneity or roughness of the crystal surface shown in Figure S12b results from larger ripples on the sample, which may be induced by small crystals or aggregations on its surface.

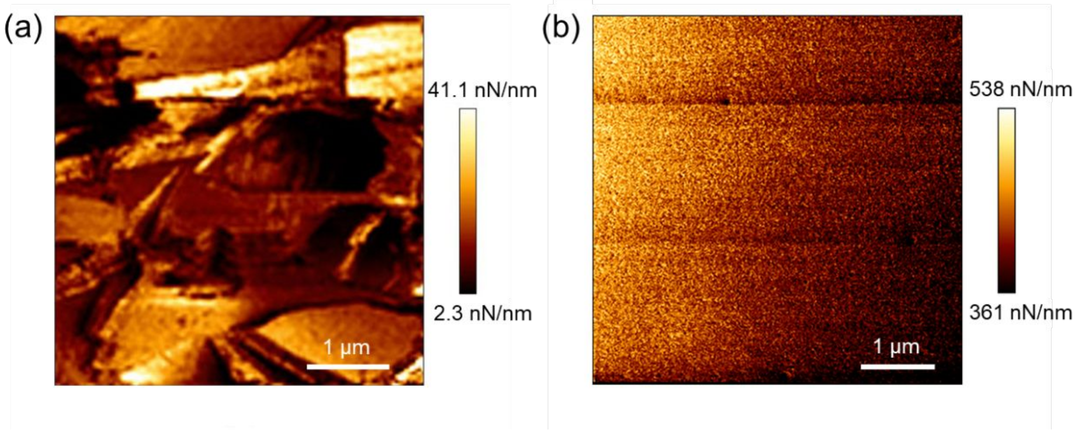

Figure S13 Statistical point stiffness of (a) cyclo-WA and (b) cyclo-WS crystals. The point stiffness data appear homogeneous in Figure S13b, indicating that the same materials exhibit similar point stiffness.

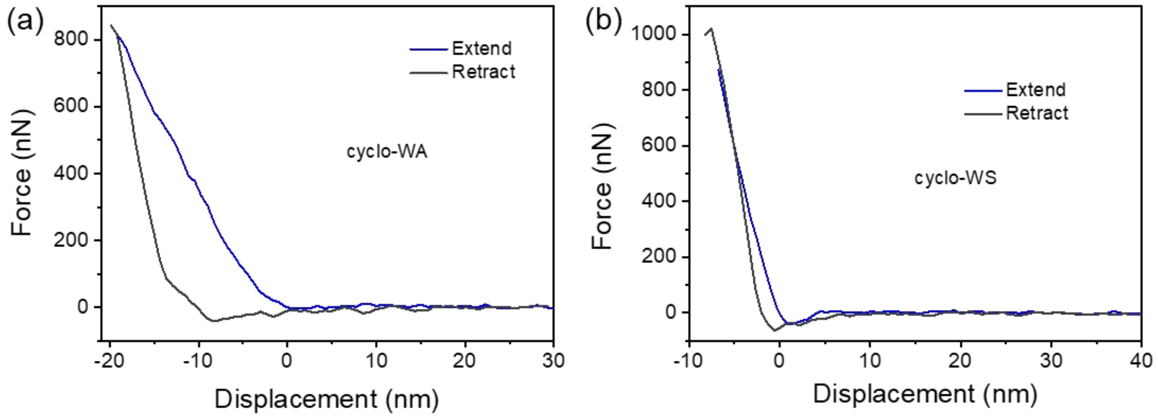

Figure S14 Typical force-displacement traces on (a) cyclo-WA and (b) cyclo-WS crystals.

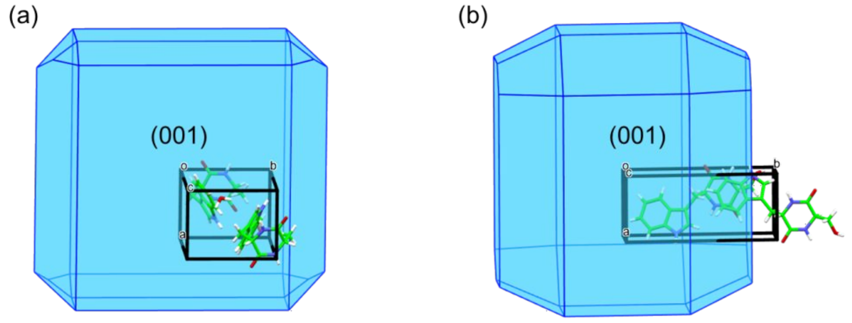

Figure S15 Predicted morphologies of (a) cyclo-WA and (b) cyclo-WS.

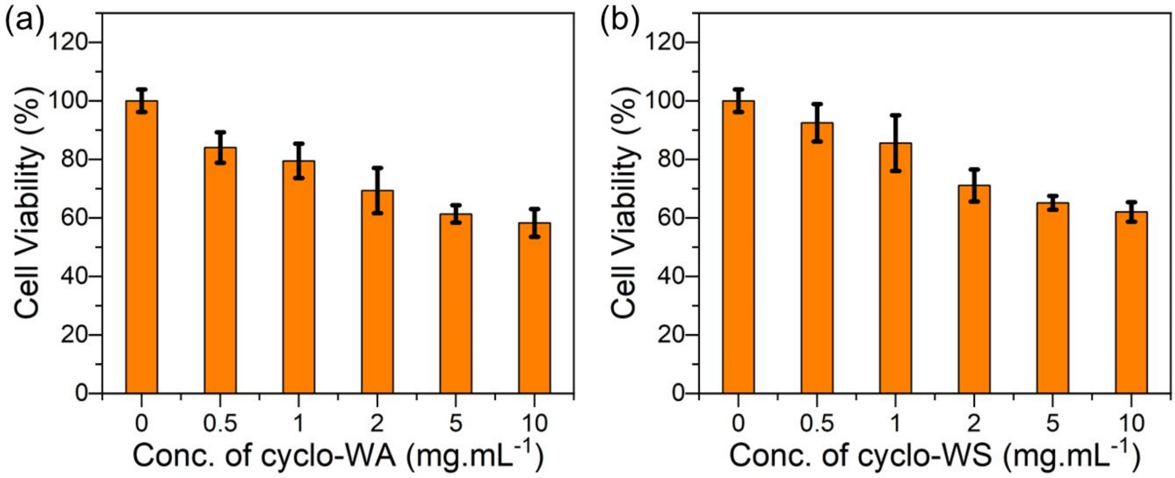

Figure S16 (a, b) MTT cell viability analysis of HeLa cells grown in the presence of (a) cyclo-WA and (b) cyclo-WS crystals at different concentrations, as indicated.

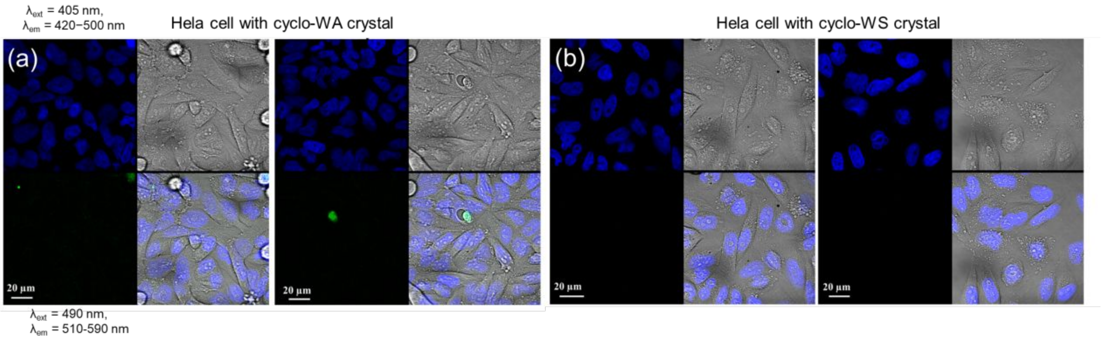

Figure S17 (a,b) Confocal fluorescence images of HeLa cells incubated with (a) cyclo-WA and (b) cyclo-WS crystals.

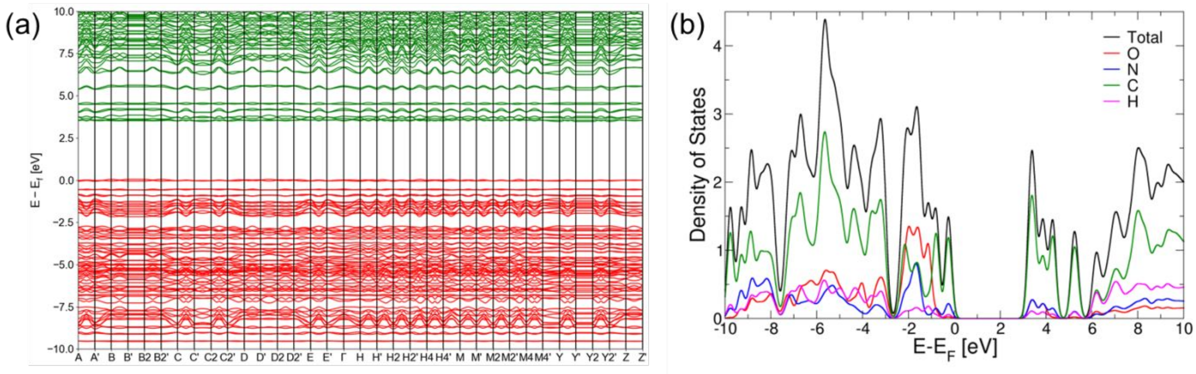

Figure S18 (a) Calculated band structure showing the showing the bandgap for cyclo-WS crystals. (b) The corresponding density of states of cyclo-WS crystal, with the Fermi energy level set to zero.

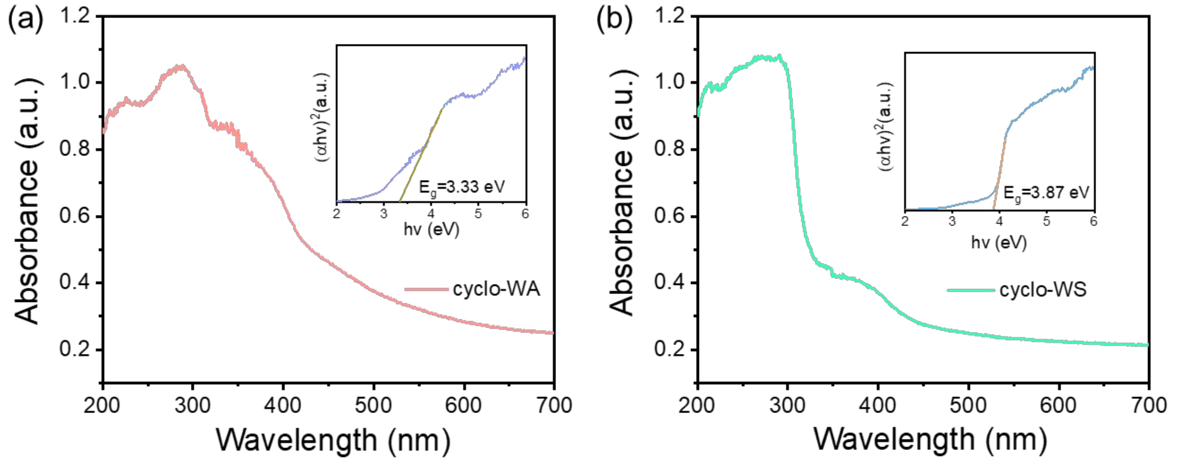

Figure S19 UV-visible absorption spectra of (a) cyclo-WA and (b) cyclo-WS. The inset shows corresponding band gaps.

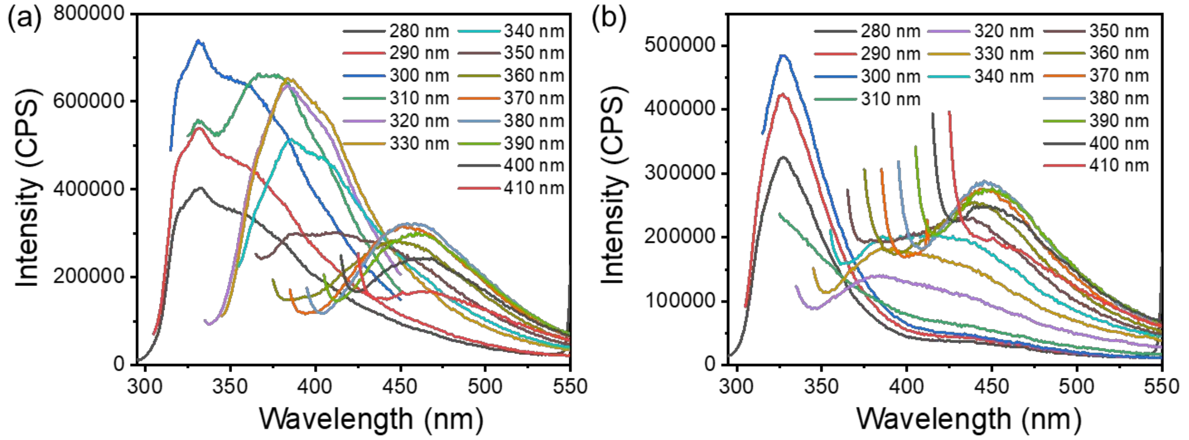

Figure S20 Fluorescence emission spectra of (a) cyclo-WA and (b) cyclo-WS at different excitations.

Table S1 Data collection and refinement statistics of cyclo-WA and cyclo-WS crystals.

Crystal data	Cyclo-WA	Cyclo-WS
CCDC Deposition number	2304390	2304391
Chemical formula	$C_{14}H_{17}N_3O_3$ ($C_{14}H_{15}N_3O_2.H_2O$)	$C_{14}H_{15}N_3O_3$
Crystal system	monoclinic	monoclinic
Space group	$P\,2_1$	$P\,2_1$
Mr	275.30	273.29
Volume (Å^3)	650.61(5)	648.019(14)
a(Å)	6.2780(3)	6.17730(10)
b (Å)	7.9007(4)	13.32090(10)
c (Å)	13.2593(6)	8.16720(10)
α (°)	90	90
β (°)	98.401(2)	105.3700(10)
γ (°)	90	90
Z	2	2
Mu (mm^{-1})	0.101	0.833
Temperature	110(2) K	113.15 K
Data collection Diffractometer	Bruker Kappa ApexDuo (Cu)	Rigaku 007HF(Cu)
No. of reflections(unique)	12691(4524)	11198 (2660)
R_{int}	0.0557	0.0418
θ° range	1.55 to 32.05°	11.236 to 154.348
Reflections/restraints/parameters	4524/1/202	2660/1/198
R[F2 > 2σ(F2)]	0.046	0.0308
wR(F2)	0.119	0.0814
Goodness-of-fit	1.074	1.122
H-atom treatment	mixed	mixed

Table S2 Calculated piezoelectric charge tensor components e_{ij} (in units of C m^{-2}), strain tensor components d_{ik} (pC V^{-1}) and piezoelectric Voltage Tensor (mV mN^{-1}) of cyclo-WA crystals.

Dielectric Constants

$$\begin{pmatrix} 2.838 & 0 & 0.049 \\ 0 & 2.651 & 0 \\ 0.049 & 0 & 2.521 \end{pmatrix}$$

Piezoelectric Charge Tensor [e] (C m^{-2})

$$\begin{pmatrix} 0 & 0 & 0 & 0.002326 & 0 & -0.000846 \\ 0.038700 & 0.083009 & -0.088721 & 0 & -0.023927 & 0 \\ 0 & 0 & 0 & -0.035333 & 0 & 0.090547 \end{pmatrix}$$

Piezoelectric Strain Tensor (pC N^{-1})

$$\begin{pmatrix} 0 & 0 & 0 & 0.491 & 0 & -0.206 \\ -0.282 & 9.332 & -7.742 & 0 & -7.159 & 0 \\ 0 & 0 & 0 & -11.607 & 0 & 10.715 \end{pmatrix}$$

Piezoelectric Voltage Tensor (mV mN^{-1})

$$\begin{pmatrix} 0 & 0 & 0 & 27.358 & 0 & -15.418 \\ -11.268 & 371.867 & -308.531 & 0 & -285.271 & 0 \\ 0 & 0 & 0 & -490.009 & 0 & 452.201 \end{pmatrix}$$

Table S3 Calculated piezoelectric charge tensor components e_{ij} (in units of C m^{-2}), strain tensor components d_{ik} (pC V^{-1}) and piezoelectric Voltage Tensor (mV mN^{-1}) of cyclo-WS crystals.

Dielectric Constants

$$\begin{pmatrix} 2.774 & 0 & 0.562 \\ 0 & 3.153 & 0 \\ 0.562 & 0 & 2.236 \end{pmatrix}$$

Piezoelectric Charge Tensor [e] (C m^{-2})

$$\begin{pmatrix} 0 & 0 & 0 & -0.004993 & 0 & 0.039539 \\ -0.021266 & 0.010672 & -0.078373 & 0 & -0.002786 & 0 \\ 0 & 0 & 0 & -0.023393 & 0 & 0.026840 \end{pmatrix}$$

Piezoelectric Strain Tensor (pC N^{-1})

$$\begin{pmatrix} 0 & 0 & 0 & -5.890 & 0 & 5.261 \\ -0.104 & 4.780 & -8.040 & 0 & -2.418 & 0 \\ 0 & 0 & 0 & -9.360 & 0 & 5.874 \end{pmatrix}$$

Piezoelectric Voltage Tensor (mV mN^{-1})

$$\begin{pmatrix} 0 & 0 & 0 & -147.260 & 0 & 159.178 \\ -3.660 & 167.069 & -280.983 & 0 & -84.526 & 0 \\ 0 & 0 & 0 & -425.479 & 0 & 249.632 \end{pmatrix}$$

REFERENCES

(1) Hutter, J.; Iannuzzi, M.; Schiffmann, F.; VandeVondele, J. Cp2k: Atomistic Simulations of Condensed Matter Systems Wiley Interdiscip. Rev. Comput. Mol. Sci. 2014, 4 (1), 15-25.

(2) Argaman, N.; Makov, G. Density Functional Theory: An Introduction. *Am. J. Phys.* **2000**, *68* (1), 69-79.

(3) VandeVondele, J.; Hutter, J. An Efficient Orbital Transformation Method for Electronic Structure Calculations. *J. Chem. Phys.* **2003**, *118* (10), 4365-4369.

(4) VandeVondele, J.; Hutter, J. Gaussian Basis Sets for Accurate Calculations on Molecular Systems in Gas and Condensed Phases. *J. Chem. Phys.* **2007**, *127* (11), 114105.

(5) Ernzerhof, M.; Scuseria, G. E. Assessment of the Perdew–Burke–Ernzerhof Exchange-Correlation Functional. *J. Chem. Phys.* **1999**, *110* (11), 5029-5036.

(6) Perdew, J. P.; Burke, K.; Ernzerhof, M. Generalized Gradient Approximation Made Simple. *Phys. Rev. Lett.* **1996**, *77* (18), 3865-3868.

(7) Grimme, S.; Ehrlich, S.; Goerigk, L. Effect of the Damping Function in Dispersion Corrected Density Functional Theory. *J. Comput. Chem.* **2011**, *32* (7), 1456-1465.

(8) Grimme, S.; Antony, J.; Ehrlich, S.; Krieg, H. A Consistent and Accurate Ab Initio Parametrization of Density Functional Dispersion Correction (Dft-D) for the 94 Elements H-Pu. *J. Chem. Phys*. **2010**, *132* (15), 154104.

(9) Štich, I.; Car, R.; Parrinello, M.; Baroni, S. Conjugate Gradient Minimization of the Energy Functional: A New Method for Electronic Structure Calculation. *Phys. Rev. B*. **1989**, *39* (8), 4997-5004.

(10) Hinuma, Y.; Pizzi, G.; Kumagai, Y.; Oba, F.; Tanaka, I. Band Structure Diagram Paths Based on Crystallography. *Comput. Mater. Sci.* **2017**, 128 140-184.
